# *N*′-[3-(Hy­droxy­imino)­butan-2-yl­idene]-4-methyl­benzene-1-sulfono­hydrazide

**DOI:** 10.1107/S1600536812003339

**Published:** 2012-02-04

**Authors:** Maria C. S. Bulhosa, Vanessa Carratu Gervini, Leandro Bresolin, Aline Locatelli, Adriano Bof de Oliveira

**Affiliations:** a Escola de Química e Alimentos, Universidade Federal do Rio Grande, Av. Itália km 08, Campus Carreiros, 96201-900, Rio Grande, RS, Brazil; b Departamento de Química, Universidade Federal de Santa Maria, Av. Roraima, Campus, 97105-900, Santa Maria, RS, Brazil; c Departamento de Química, Universidade Federal de Sergipe, Av. Marechal Rondon s/n, Campus, 49100-000, São Cristóvão, SE, Brazil

## Abstract

In the title compound, C_11_H_15_N_3_O_3_S, the C—S—N(H)—N linkage is nonplanar, the torsion angle being 75.70 (12)°. The compound has two almost planar fragments linked to the S atom: the hydrazone-derivative fragment [(HONC_4_H_6_)N—N(H)–] and the tolyl fragment (C_7_H_7_–) have maximum deviations from the mean plane through the non-H atoms of 0.0260 (10) and 0.0148 (14) Å, respectively. The two planar fragments make an inter­planar angle of 79.47 (5)°. In the crystal, mol­ecules are connected through inversion centers *via* pairs of N—H⋯O and O—H⋯N hydrogen bonds.

## Related literature

For the synthesis and application of hy­droxy­imino-tosyl­hydrazones as complexing agents, see: Beger *et al.* (1991 ▶). For a similar structure with a tosyl­hydrazone derivative, see: Fonseca *et al.* (2011 ▶).
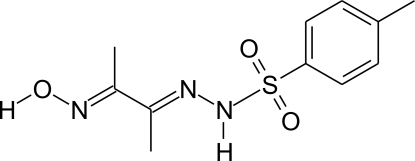


## Experimental

### 

#### Crystal data

C_11_H_15_N_3_O_3_S
*M* *_r_* = 269.32
Triclinic, 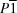

*a* = 5.5740 (1) Å
*b* = 10.4354 (2) Å
*c* = 11.3997 (2) Å
α = 83.586 (1)°
β = 77.453 (1)°
γ = 87.688 (1)°
*V* = 643.11 (2) Å^3^
*Z* = 2
Mo *K*α radiation
μ = 0.26 mm^−1^
*T* = 293 K
0.55 × 0.24 × 0.22 mm

#### Data collection

Bruker APEXII CCD diffractometer
Absorption correction: multi-scan (*SADABS*; Bruker, 2005 ▶) *T*_min_ = 0.872, *T*_max_ = 0.946
11000 measured reflections
3211 independent reflections
2862 reflections with *I* > 2σ(*I*)
*R*_int_ = 0.015

#### Refinement

*R*[*F* ^2^ > 2σ(*F* ^2^)] = 0.036
*wR*(*F* ^2^) = 0.112
*S* = 1.05
3211 reflections
174 parameters
H atoms treated by a mixture of independent and constrained refinement
Δρ_max_ = 0.27 e Å^−3^
Δρ_min_ = −0.28 e Å^−3^

### 

Data collection: *COSMO* (Bruker, 2005 ▶); cell refinement: *SAINT*; data reduction: *SAINT* (Bruker, 2005 ▶); program(s) used to solve structure: *SHELXS97* (Sheldrick, 2008 ▶); program(s) used to refine structure: *SHELXL97* (Sheldrick, 2008 ▶); molecular graphics: *DIAMOND* (Brandenburg, 2006 ▶); software used to prepare material for publication: *publCIF* (Westrip, 2010 ▶).

## Supplementary Material

Crystal structure: contains datablock(s) I, global. DOI: 10.1107/S1600536812003339/fy2042sup1.cif

Structure factors: contains datablock(s) I. DOI: 10.1107/S1600536812003339/fy2042Isup2.hkl

Supplementary material file. DOI: 10.1107/S1600536812003339/fy2042Isup3.cml

Additional supplementary materials:  crystallographic information; 3D view; checkCIF report

## Figures and Tables

**Table 1:** Hydrogen-bond geometry (Å, °)

*D*—H⋯*A*	*D*—H	H⋯*A*	*D*⋯*A*	*D*—H⋯*A*
N3—H8⋯O3^i^	0.82 (2)	2.19 (2)	2.9830 (18)	165.0 (19)
O1—H1⋯N1^ii^	0.84 (3)	1.99 (3)	2.792 (2)	160 (2)

Symmetry codes: (i) 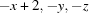; (ii) 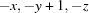.

**Fig. 1.**
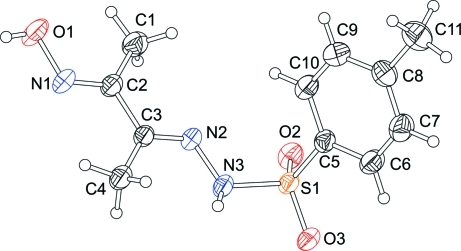
The molecular structure of the title compound with labeling and displacement ellipsoids drawn at the 40% probability level.

**Fig. 2.**
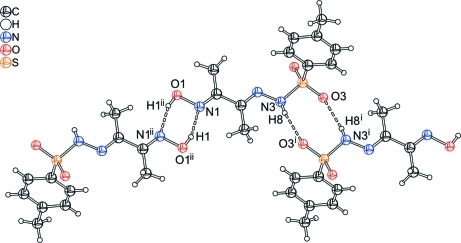
Molecules of the title compound connected through inversion centers via pairs of N—H···O and O—H···N hydrogen bonds in the crystal structure. Intermolecular hydrogen bonding is indicated as dashed lines. Symmetry codes: (i)-x+2, -y, -z; (ii)-x, -y+1, -z.

## supplementary crystallographic information

### Comment

Hydrazones have a wide range of applications on inorganic chemistry. For example, sulfonylhydrazones are used as complexing agents for cobalt (II) (Beger *et al.*, 1991). As part of our study of sulfonylhydrazone derivatives, we report herein the crystal structure of an oxime-sulfonylhydrazone derivative. In the title compound (Fig. 1) the C—S—N(H)—N linkage is non-planar with the torsion angle being 75.70 (12)° and a tetrahedral environment suggests a *sp*^3^ hybridization for the S atom. The title structure contains additionally two planar fragments. The maximum deviations from the least squares planes for the hydrazone derivative fragment C1/C2/C3/C4/N1/N2/N3/O1 and for the tolyl fragment C5/C6/C7/C8/C9/C10/C11 amount to 0.0260 (10)° and for N3 and 0.0148 (14)° for C9 atoms, respectively. The dihedral angle between the two planes is 79.47 (5)°. The crystal packing is stabilized by intermolecular N—H···O (Table 1; N3—H8···O3^i^) and O—H···N bonds (Table 1; O1—H1···N1^ii^) connecting the molecules through inversion centers (Fig. 2). Symmetry codes: (i)*-x*+2, *-y*, *-z*; (ii)*-x*, *-y*+1, *-z*.

### Experimental

Starting materials were commercially available and were used without further purification. The synthesis was adapted from a procedure reported previously (Beger *et al.*, 1991). The reaction of 2,3-Butanedione monoxime (5 mmol) and *p*-toluenesulfonylhydrazine (5 mmol) in ethanol (50 ml) was refluxed for 3 h. After cooling and filtering, crystals suitable for X-ray diffraction were obtained.

### Refinement

H atoms attached to C atoms were positioned with idealized geometry and were refined isotropically with *U*_iso_(H) set to 1.2 times *U*_eq_(C) for the aromatic and 1.5 times *U*_eq_(C) for methyl H atoms using a riding model with C—H = 0.93 Å and C—H = 0.96 Å, respectively. H atoms attached to N and O atoms were located in difference Fourier maps and included in the subsequent refinement using restraints (N3—H8 = 0.82 (2) Å and O1—H1 = 0.84 (3) Å) with *U*_iso_(H) = 1.5 times of the *U*_eq_(N) and *U*_eq_(O), respectively. In the last stage of refinement, they were refined freely.

### Crystal data

**Table:**

C_11_H_15_N_3_O_3_S	*Z* = 2
*M**_r_* = 269.32	*F*(000) = 284
Triclinic, *P*1	*D*_x_ = 1.391 Mg m^−^^3^
Hall symbol: -P 1	Mo *K*α radiation, λ = 0.71073 Å
*a* = 5.5740 (1) Å	Cell parameters from 6049 reflections
*b* = 10.4354 (2) Å	θ = 2.6–28.3°
*c* = 11.3997 (2) Å	µ = 0.26 mm^−^^1^
α = 83.586 (1)°	*T* = 293 K
β = 77.453 (1)°	Block, colourless
γ = 87.688 (1)°	0.55 × 0.24 × 0.22 mm
*V* = 643.11 (2) Å^3^	

### Data collection

**Table:**

Bruker APEXII CCD diffractometer	3211 independent reflections
Radiation source: fine-focus sealed tube, Bruker X8 APEXII	2862 reflections with *I* > 2σ(*I*)
Graphite monochromator	*R*_int_ = 0.015
φ and ω scans	θ_max_ = 28.3°, θ_min_ = 1.8°
Absorption correction: multi-scan (*SADABS*; Bruker, 2005)	*h* = −7→6
*T*_min_ = 0.872, *T*_max_ = 0.946	*k* = −13→13
11000 measured reflections	*l* = −15→15

### Refinement

**Table:**

Refinement on *F*^2^	Primary atom site location: structure-invariant direct methods
Least-squares matrix: full	Secondary atom site location: difference Fourier map
*R*[*F*^2^ > 2σ(*F*^2^)] = 0.036	Hydrogen site location: inferred from neighbouring sites
*wR*(*F*^2^) = 0.112	H atoms treated by a mixture of independent and constrained refinement
*S* = 1.05	*w* = 1/[σ^2^(*F*_o_^2^) + (0.0651*P*)^2^ + 0.1526*P*] where *P* = (*F*_o_^2^ + 2*F*_c_^2^)/3
3211 reflections	(Δ/σ)_max_ < 0.001
174 parameters	Δρ_max_ = 0.27 e Å^−^^3^
0 restraints	Δρ_min_ = −0.28 e Å^−^^3^

### Special details

**Table:**

Geometry. All e.s.d.'s (except the e.s.d. in the dihedral angle between two l.s. planes) are estimated using the full covariance matrix. The cell e.s.d.'s are taken into account individually in the estimation of e.s.d.'s in distances, angles and torsion angles; correlations between e.s.d.'s in cell parameters are only used when they are defined by crystal symmetry. An approximate (isotropic) treatment of cell e.s.d.'s is used for estimating e.s.d.'s involving l.s. planes.
Refinement. Refinement of *F*^2^ against ALL reflections. The weighted *R*-factor *wR* and goodness of fit *S* are based on *F*^2^, conventional *R*-factors *R* are based on *F*, with *F* set to zero for negative *F*^2^. The threshold expression of *F*^2^ > σ(*F*^2^) is used only for calculating *R*-factors(gt) *etc*. and is not relevant to the choice of reflections for refinement. *R*-factors based on *F*^2^ are statistically about twice as large as those based on *F*, and *R*- factors based on ALL data will be even larger.

### Fractional atomic coordinates and isotropic or equivalent isotropic displacement parameters (Å^2^)

**Table:**

	*x*	*y*	*z*	*U*_iso_*/*U*_eq_	
S1	0.94931 (6)	0.06163 (3)	0.19318 (3)	0.03670 (12)	
O3	1.14412 (19)	−0.00383 (11)	0.11725 (10)	0.0465 (3)	
O1	0.0184 (2)	0.53370 (13)	0.13328 (14)	0.0599 (3)	
O2	1.00547 (19)	0.14436 (11)	0.27426 (10)	0.0474 (3)	
N3	0.8101 (2)	0.14880 (12)	0.09796 (12)	0.0410 (3)	
N2	0.6390 (2)	0.23836 (11)	0.14677 (11)	0.0383 (3)	
N1	0.1759 (2)	0.44076 (12)	0.07432 (12)	0.0446 (3)	
C3	0.4937 (2)	0.28939 (12)	0.08064 (12)	0.0361 (3)	
C5	0.7414 (2)	−0.05473 (13)	0.27630 (13)	0.0377 (3)	
C2	0.3235 (2)	0.38812 (13)	0.13806 (13)	0.0393 (3)	
C8	0.4212 (3)	−0.23753 (16)	0.41661 (14)	0.0489 (4)	
C6	0.8088 (3)	−0.18421 (15)	0.28051 (16)	0.0493 (4)	
H9	0.9602	−0.2102	0.2366	0.059*	
C9	0.3564 (3)	−0.10782 (18)	0.40921 (16)	0.0546 (4)	
H11	0.2030	−0.0821	0.4513	0.066*	
C10	0.5143 (3)	−0.01583 (16)	0.34077 (16)	0.0502 (4)	
H12	0.4691	0.0710	0.3379	0.060*	
C4	0.4871 (3)	0.25726 (17)	−0.04261 (15)	0.0496 (4)	
H5	0.4554	0.1671	−0.0395	0.074*	
H6	0.6424	0.2770	−0.0962	0.074*	
H7	0.3591	0.3070	−0.0716	0.074*	
C7	0.6482 (3)	−0.27425 (16)	0.35074 (17)	0.0554 (4)	
H10	0.6934	−0.3611	0.3539	0.066*	
C11	0.2476 (4)	−0.3351 (2)	0.49484 (18)	0.0665 (5)	
H13	0.0895	−0.3248	0.4741	0.100*	
H14	0.2317	−0.3220	0.5782	0.100*	
H15	0.3110	−0.4205	0.4820	0.100*	
C1	0.3317 (3)	0.41963 (17)	0.26113 (15)	0.0539 (4)	
H2	0.1975	0.4771	0.2887	0.081*	
H3	0.4844	0.4604	0.2586	0.081*	
H4	0.3186	0.3418	0.3155	0.081*	
H8	0.794 (4)	0.1114 (19)	0.0409 (19)	0.053 (5)*	
H1	−0.061 (5)	0.556 (2)	0.079 (2)	0.082 (8)*	

### Atomic displacement parameters (Å^2^)

**Table:**

	*U*^11^	*U*^22^	*U*^33^	*U*^12^	*U*^13^	*U*^23^
S1	0.03284 (18)	0.03859 (19)	0.0401 (2)	0.00890 (12)	−0.01154 (13)	−0.00617 (13)
O3	0.0385 (5)	0.0504 (6)	0.0492 (6)	0.0159 (4)	−0.0088 (4)	−0.0071 (5)
O1	0.0532 (7)	0.0575 (7)	0.0732 (8)	0.0290 (6)	−0.0195 (6)	−0.0236 (6)
O2	0.0453 (5)	0.0482 (6)	0.0538 (6)	0.0033 (4)	−0.0191 (5)	−0.0124 (5)
N3	0.0415 (6)	0.0419 (6)	0.0407 (6)	0.0153 (5)	−0.0128 (5)	−0.0067 (5)
N2	0.0352 (5)	0.0364 (6)	0.0425 (6)	0.0083 (4)	−0.0080 (5)	−0.0040 (5)
N1	0.0374 (6)	0.0396 (6)	0.0567 (8)	0.0137 (5)	−0.0106 (5)	−0.0093 (5)
C3	0.0323 (6)	0.0333 (6)	0.0412 (7)	0.0042 (5)	−0.0067 (5)	−0.0015 (5)
C5	0.0373 (6)	0.0396 (7)	0.0382 (7)	0.0059 (5)	−0.0133 (5)	−0.0053 (5)
C2	0.0347 (6)	0.0357 (6)	0.0461 (7)	0.0045 (5)	−0.0064 (5)	−0.0045 (6)
C8	0.0541 (8)	0.0520 (9)	0.0427 (8)	−0.0057 (7)	−0.0148 (6)	−0.0042 (7)
C6	0.0467 (8)	0.0431 (8)	0.0566 (9)	0.0095 (6)	−0.0073 (7)	−0.0096 (7)
C9	0.0450 (8)	0.0599 (10)	0.0548 (9)	0.0056 (7)	−0.0030 (7)	−0.0059 (8)
C10	0.0460 (8)	0.0444 (8)	0.0568 (9)	0.0110 (6)	−0.0060 (7)	−0.0041 (7)
C4	0.0522 (8)	0.0521 (9)	0.0457 (8)	0.0189 (7)	−0.0145 (7)	−0.0094 (7)
C7	0.0620 (10)	0.0392 (8)	0.0640 (10)	0.0038 (7)	−0.0123 (8)	−0.0053 (7)
C11	0.0719 (12)	0.0650 (11)	0.0590 (11)	−0.0142 (9)	−0.0079 (9)	0.0007 (9)
C1	0.0585 (9)	0.0545 (9)	0.0503 (9)	0.0140 (7)	−0.0129 (7)	−0.0153 (7)

### Geometric parameters (Å, º)

**Table:**

S1—O2	1.4225 (11)	C8—C11	1.506 (2)
S1—O3	1.4349 (10)	C6—C7	1.383 (2)
S1—N3	1.6420 (12)	C6—H9	0.9300
S1—C5	1.7591 (14)	C9—C10	1.381 (2)
O1—N1	1.4084 (16)	C9—H11	0.9300
O1—H1	0.84 (3)	C10—H12	0.9300
N3—N2	1.3807 (16)	C4—H5	0.9600
N3—H8	0.82 (2)	C4—H6	0.9600
N2—C3	1.2843 (18)	C4—H7	0.9600
N1—C2	1.2808 (19)	C7—H10	0.9300
C3—C2	1.4806 (18)	C11—H13	0.9600
C3—C4	1.489 (2)	C11—H14	0.9600
C5—C6	1.386 (2)	C11—H15	0.9600
C5—C10	1.389 (2)	C1—H2	0.9600
C2—C1	1.487 (2)	C1—H3	0.9600
C8—C9	1.385 (2)	C1—H4	0.9600
C8—C7	1.387 (2)		
			
O2—S1—O3	119.84 (7)	C10—C9—C8	121.49 (15)
O2—S1—N3	107.87 (7)	C10—C9—H11	119.3
O3—S1—N3	104.20 (6)	C8—C9—H11	119.3
O2—S1—C5	108.48 (7)	C9—C10—C5	119.14 (15)
O3—S1—C5	108.09 (7)	C9—C10—H12	120.4
N3—S1—C5	107.77 (6)	C5—C10—H12	120.4
N1—O1—H1	98.5 (17)	C3—C4—H5	109.5
N2—N3—S1	116.06 (10)	C3—C4—H6	109.5
N2—N3—H8	122.1 (14)	H5—C4—H6	109.5
S1—N3—H8	113.8 (14)	C3—C4—H7	109.5
C3—N2—N3	117.27 (12)	H5—C4—H7	109.5
C2—N1—O1	112.69 (13)	H6—C4—H7	109.5
N2—C3—C2	113.63 (13)	C6—C7—C8	121.31 (15)
N2—C3—C4	125.81 (13)	C6—C7—H10	119.3
C2—C3—C4	120.56 (12)	C8—C7—H10	119.3
C6—C5—C10	120.45 (14)	C8—C11—H13	109.5
C6—C5—S1	119.79 (11)	C8—C11—H14	109.5
C10—C5—S1	119.72 (11)	H13—C11—H14	109.5
N1—C2—C3	115.11 (13)	C8—C11—H15	109.5
N1—C2—C1	124.73 (13)	H13—C11—H15	109.5
C3—C2—C1	120.15 (12)	H14—C11—H15	109.5
C9—C8—C7	118.35 (15)	C2—C1—H2	109.5
C9—C8—C11	120.18 (16)	C2—C1—H3	109.5
C7—C8—C11	121.47 (16)	H2—C1—H3	109.5
C7—C6—C5	119.24 (15)	C2—C1—H4	109.5
C7—C6—H9	120.4	H2—C1—H4	109.5
C5—C6—H9	120.4	H3—C1—H4	109.5
			
O2—S1—N3—N2	−41.25 (12)	N2—C3—C2—N1	−179.95 (12)
O3—S1—N3—N2	−169.63 (10)	C4—C3—C2—N1	0.2 (2)
C5—S1—N3—N2	75.70 (12)	N2—C3—C2—C1	0.0 (2)
S1—N3—N2—C3	−166.53 (10)	C4—C3—C2—C1	−179.93 (15)
N3—N2—C3—C2	−177.60 (11)	C10—C5—C6—C7	−0.7 (2)
N3—N2—C3—C4	2.3 (2)	S1—C5—C6—C7	176.99 (13)
O2—S1—C5—C6	−117.69 (13)	C7—C8—C9—C10	−1.6 (3)
O3—S1—C5—C6	13.69 (15)	C11—C8—C9—C10	178.28 (17)
N3—S1—C5—C6	125.76 (13)	C8—C9—C10—C5	1.1 (3)
O2—S1—C5—C10	60.01 (14)	C6—C5—C10—C9	0.0 (2)
O3—S1—C5—C10	−168.61 (12)	S1—C5—C10—C9	−177.65 (13)
N3—S1—C5—C10	−56.54 (14)	C5—C6—C7—C8	0.2 (3)
O1—N1—C2—C3	179.96 (12)	C9—C8—C7—C6	0.9 (3)
O1—N1—C2—C1	0.1 (2)	C11—C8—C7—C6	−178.96 (17)

### Hydrogen-bond geometry (Å, º)

**Table:**

*D*—H···*A*	*D*—H	H···*A*	*D*···*A*	*D*—H···*A*
N3—H8···O3^i^	0.82 (2)	2.19 (2)	2.9830 (18)	165.0 (19)
O1—H1···N1^ii^	0.84 (3)	1.99 (3)	2.792 (2)	160 (2)

Symmetry codes: (i) −*x*+2, −*y*, −*z*; (ii) −*x*, −*y*+1, −*z*.
